# Post-BSSO condylar position stability: a comparison of miniplate and lag screw fixation

**DOI:** 10.1186/s12903-024-04499-w

**Published:** 2024-06-25

**Authors:** Ehsan Aliabadi, Fateme Eskandari, Milad Zanjani, Moslem Babouei

**Affiliations:** 1 Department of Oral and Maxillofacial Surgery, School of Dentistry, Shiraz University of Medical Sciences, Shiraz, 71956-15878 Iran; 2 School of Dentistry, Shiraz University of Medical Sciences, Ghasrdasht Street, Shiraz, 71956-15878 Iran

**Keywords:** Facial asymmetry, Mandibular condyle, Maxillofacial surgery, Osteotomy, Sagittal split ramus

## Abstract

**Background:**

This study was conceived to assess the postoperative stability of condylar position following fixation with miniplates and lag screws after bilateral sagittal split osteotomy (BSSO).

**Methods:**

This retrospective study included a cohort of 20 patients undergoing BSSO using the Obwegeser-Dal Pont modification. The bony segments were stabilized using either miniplates with two 2.0-mm monocortical screws per segment or three 2.0-mm bicortical lag screws along the mandible’s superior border. Pre- and postoperative (7-day interval) spiral computed tomography scans were conducted to assess skeletal changes across both groups. Data analysis employed Wilcoxon signed-rank and Wilcoxon rank-sum tests (α = 0.05).

**Results:**

No statistically significant difference was observed between the pre-and postoperative condylar position parameters (P>0.05). However, the lag screw group showed a marginal significant increase in the left condyle’s angulation (preoperative: 24.83 ± 6.37 vs. postoperative: 32.5 ± 4.93; *P* = 0.04). Changes in condylar height, length, and width were not statistically significant before and after BSSO in either groups (P>0.05). Nor was any statistically significant difference found between the miniplates and lag screws groups regarding condylar position parameters (P>0.05).

**Conclusion:**

The results indicated that both lag screw and miniplate fixation methods can be effectively employed in BSSO procedures without impacting condylar position parameters. Thus, either fixation method can be chosen depending on factors such as the surgeon’s preference and clinical outcomes.

## Background

Facial asymmetry represents a prevalent dentofacial deformity, often manifesting in the maxilla, mandible, or chin across the horizontal, vertical, and transverse planes [1]. Bilateral sagittal split osteotomy (BSSO), with or without Le Fort I osteotomy, has emerged as the predominant orthognathic surgical intervention to address facial asymmetry [2–4]. In this surgical procedure, the mandible is divided into distal and proximal segments to facilitate controlled displacement. Meanwhile, preserving the original positions of the condylar heads holds substantial importance, as it contributes to averting a relapse [5].

To facilitate effective repair, the osteotomized segments should be fixed through various techniques and tools such as metal plates and/or screws. Particularly, stable internal fixation involves positioning these components directly in contact with the bone, enabling them to contribute to the repair process, thus minimizing the need for maxillomandibular blocks. This approach requires different sizes and types of instruments such as positional or compressive screws, monocortical plates, or their combination [6, 7]. Bicortical or monocortical screws and plates are the most-commonly used instruments to reapproximate the two osteotomized fragments [8].

An approach to stabilize the proximal and distal segments is through rigid internal fixation with multiple bicortical positional screws, which expedites bone healing and mandibular function recovery, obviates postoperative intermaxillary fixation, and minimizes postoperative relapse. An alternative post-BSSO fixation method is semirigid fixation with monocortical miniplates. This method allows intraoral access without the transbuccal approach, as well as, facile adjustment of the proximal segment and condyle position peri- and postoperatively. However, in vitro evidence suggests that miniplate fixation exhibit lower mechanical stability compared to bicortical screw fixation [9]. Clinical reports have documented instances of miniplate bending or breakage [10, 11]. Conversely, numerous in-vitro and comparative clinical studies showed that postoperative changes do not differ significantly between bicortical screw fixation and monocortical miniplate fixation techniques [12].

While numerous studies have examined the stability of condylar position and the influence of fixation methods following BSSO, the existing literature reveals some notable limitations. Also, the majority of the existing literature has focused on either a single fixation method or a comparison to other techniques. Given the substantial concern surrounding postoperative relapse in orthognathic surgery [12–14] and paucity of literature, the current study was designed to assess the post-BSSO stability of condylar position following fixation with miniplates and lag screws.

## Methods

### Study design and participants

The study design was approved by the Ethics Committee of Shiraz University of Medical Sciences, Shiraz, Iran (IR.SUMS.DENTAL.REC.1400.038). It was performed in full accordance with ethical principles, including the World Medical Association Declaration of Helsinki (version 2008). Written informed consent was obtained from all patients.

The study included 20 Iranian adults (11 males and 9 females) under 30 years old, referred to Rajaei Hospital’s oral and maxillofacial surgery department from November 2020 to November 2022. The inclusion criteria were mandibular retrognathism with/without facial asymmetry, indication of mandibular advancement BSSO with/without Le Fort I osteotomy or genioplasty, available preoperative/postoperative spiral computed tomography (CT) images, maximum abnormality of 7 mm, and no history of trauma or craniofacial syndrome (such as cleft lip and palate). The exclusion criteria of this study were patients who require surgical movements of more than 7 mm in any planes, midline shifts of more than 5 mm, any form of facial clefts, hemifacial macrosomia, Pierre Robin sequence, Treacher-Collin syndrome, mental disorders, severe facial asymmetry, and those showing clinical symptoms of temporomandibular disorder and degenerative joint disease.

### Surgical procedures

Each patient received orthodontic therapy before and after their operation. BSSO of the ramus was performed using the Obwegeser-Dal Pont modification along with 1-segment Le Fort I osteotomy. The maxilla was stabilized through rigid fixation with titanium miniplates and screws. There was no case of inferior repositioning of the maxilla among the treated patients. The pterygomasseteric sling of the mandible’s proximal segment was dissected at its inferior and posterior borders. The bony segments were fixed through using a miniplate to install two 2.0-mm monocortical screws in each segment (miniplate group, *n* = 10) or inserting three 2.0-mm bicortical screws at the mandible’s superior border (lag screw group, *n* = 10). Intermaxillary fixation was applied for 3 to 5 days to maintain the postoperative occlusion. Subsequently, physiotherapy instructions were provided including mouth-opening exercises with orthodontic elastics. Postoperative orthodontic treatment started one month after the surgery.

### Data acquisition

**Fig. 1:**
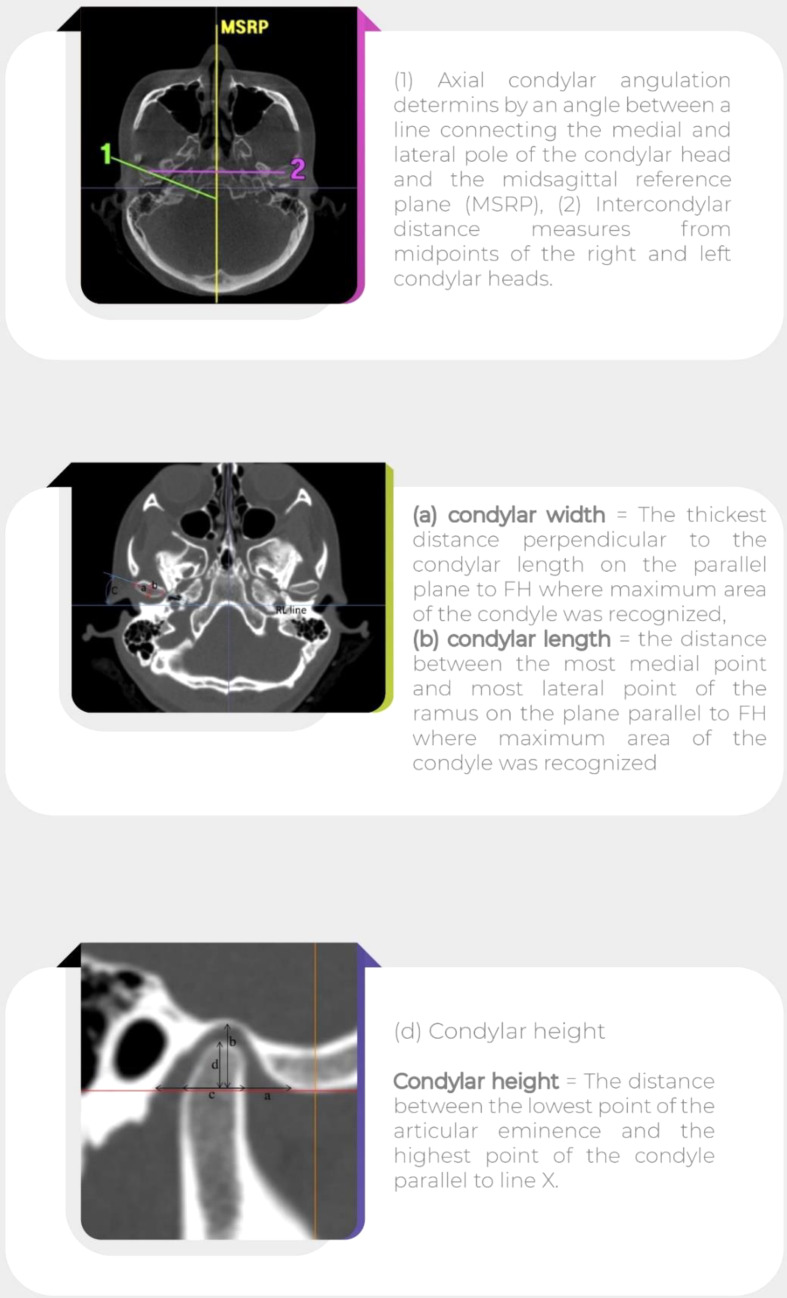
Measurement and assessment of the skeletal alterations and parameters

Spiral CT images (Somatom Definition Flash, Siemens, Germany, 120 kV, 282 mA and 26.3 s scan time) were taken preoperatively and one week postoperatively. The patients were positioned upright in maximum intercuspation, aligning the Frankfort horizontal plane with the floor. The spiral CT data was reconstructed with 3D imaging software (version 3.3.1 for Windows) to measure and assess the skeletal alterations and parameters (Fig. 1). All measurements were done using picture archiving and communication system (PACS) filmless radiology software (Version. 4, INFINITT, North America Inc., Phillipsburg, NJ, USA) with 0.1-mm accuracy.

The axial condylar angulation was measured at the widest condylar width medio- laterally in the axial cross-section of CT. At this CT section, a line connected the most medial point of each mandibular condyle to the most lateral point of each condyle. A second line was drawn from the mid-point of internal occipital protuberance through the mid-point of the base of the skull and then to the nasal septum. This line was the middle sagittal reference plane (MSRP). The angle between each condyle medio-lateral line with MSRP was measured as axial condylar angulation. At the same point in CT, but in sagittal view, the most inferior point of articular eminence was determined. A horizontal and vertical line was drawn from this point. The most superior point of condyle to horizontal line determined as condylar height (d). Distance between the most posterior part of condyle to the most anterior part of condyle measured as condylar length. Superior condylar space measured from the horizontal line to the most inferior point of glenoid fossa. Medial and lateral condylar space were measured from the most anterior and posterior points of condyle to posterior part of articular eminence and anterior part of posterior bony part of glenoid fossa, respectively.

### Statistical analysis

Data analysis was done via the R programing language (version 3.3.1 for Windows) with deducer Graphical User Interface package. The data were presented as mean ± standard deviation (SD). The distribution of the data was evaluated for normality using the Shapiro-Wilk test. The results indicated that the data did not follow a normal distribution (*p* < 0.05) for several of the key outcome variables, including condylar angulation, height, length, and width. Given the non-normal distribution of the data, we elected to use non-parametric statistical tests to compare the pre- and postoperative measurements, as well as the differences between the miniplate and lag screw fixation groups. Specifically, the Wilcoxon signed-rank test was employed to assess the within-group changes from pre- to postoperative time points, as this test does not assume normality of the data. Similarly, the Wilcoxon rank-sum test was used to compare the outcome measures between the two fixation groups, as this non-parametric test is appropriate for independent samples with non-normal distributions. The choice of these non-parametric tests was made to ensure the validity of the statistical inferences, given the observed deviations from normality in the dataset. Therefore, Wilcoxon signed-rank test and Wilcoxon rank-sum test were used for data analysis, with the statistical significance level set at P < 0.05.

## Results

All 20 enrolled patients completed the study. The patients’ mean age was 23 y/o in the miniplate and 26 y/o in the lag screw group. The miniplate group included 6 males and 4 females; while the lag screw group comprised 4 males and 6 females. In the present study, the amount of movement in either plane ranges from 3 to 7 mm. One had mandible-only advancement surgery by BSSO, and two cases had genioplasty.

No statistically significant difference was noted between the preoperative measures and all condylar position parameters in the lag screw (P>0.05) (Table 1). However, there was a slight increase in the left condyle’s angulation within the lag screw group, showing marginal significance (preoperative measure: 24.83 ± 6.37 vs. postoperative measure: 32.5 ± 4.93; *P* = 0.04).

**Table 1:** Comparing the condylar position parameters before and after BSSO in the lag screw fixation group

Parameter	Pre-operative	Post-operative	*P* value
Right side condylar height *(mm)*	1.15 ± 0.35	1.2 ± 0.32	0.4
Left side condylar height	1.27 ± 0.26	1.27 ± 0.28	0.84
Right side condylar length	1.84 ± 0.25	1.84 ± 0.21	1
Left side condylar length	1.94 ± 0.5	1.86 ± 0.25	0.84
Right side condylar width	0.77 ± 0.13	0.76 ± 0.12	1
Left side condylar width	0.79 ± 0.06	0.78 ± 0.05	0.4
Right side condyle superior space	2.43 ± 0.81	2.78 ± 0.72	0.22
Left side condyle superior space	2.51 ± 0.62	3.29 ± 1.32	0.16
Right side condyle medial space	2.6 ± 0.47	3.53 ± 1.28	0.31
Left side condyle medial space	2.55 ± 0.64	4.01 ± 1.64	0.12
Right side condyle lateral space	1.66 ± 0.41	1.78 ± 0.38	0.06
Left side condyle lateral space	1.68 ± 0.33	1.76 ± 0.15	0.44
Right side condyle angulation *(degree)*	24.83 ± 5.64	23 ± 9.8	0.75
Left side condyle angulation	24.83 ± 6.37	32.5 ± 4.93	0.04

No significant differences were found in the condylar position parameters compared to preoperative measurements within the miniplate fixation group (Table 2).

**Table 2:** Comparing the condylar position parameters before and after BSSO in the miniplate fixation group

Parameter	Pre-op	Post-op	*p*-value
Right side condylar height *(mm)*	1.49 ± 0.41	1.49 ± 0.32	1
Left side condylar height	1.54 ± 0.29	1.57 ± 0.32	0.563
Right side condylar length	1.79 ± 0.23	1.77 ± 0.22	0.4
Left side condylar length	1.74 ± 0.19	1.71 ± 0.17	0.4
Right side condylar width	0.83 ± 0.09	0.82 ± 0.11	0.789
Left side condylar width	0.8 ± 0.15	0.8 ± 0.13	1
Right side condyle superior space	2.8 ± 0.56	2.58 ± 0.67	1
Left side condyle superior space	3.05 ± 0.95	3.2 ± 1.1	0.844
Right side condyle medial space	2.67 ± 0.27	3.83 ± 0.45	0.063
Left side condyle medial space	2.7 ± 0.73	3.82 ± 1.57	0.063
Right side condyle lateral space	2.3 ± 0.31	2.36 ± 0.2	0.437
Left side condyle lateral space	2.06 ± 0.16	2.26 ± 0.29	0.094
Right side condyle angulation *(degree)*	23.67 ± 8	23.3 ± 6.8	0.293
Left side condyle angulation	27 ± 7	27 ± 7.4	1

The alterations of condylar height, length, and width were not statistically significant before and after BSSO in either group (P>0.05) (Table 3).

**Table 3:** Comparing the condylar position parameters differences (Δ) between the miniplate and lag screw fixation groups

Parameters	Lag screw	Miniplate	*P* value
Right side condylar height *(mm)*	0.05 ± 0.16	-0.002 ± 0.026	0.334
Left side condylar height	0 ± 0.17	0.028 ± 0.067	0.485
Right side condylar length	0.008 ± 0.06	-0.018 ± 0.041	0.686
Left side condylar length	-0.085 ± 0.31	-0.025 ± 0.054	0.688
Right side condylar width	-0.002 ± 0.034	-0.005 ± 0.036	0.871
Left side condylar width	-0.013 ± 0.36	0.003 ± 0.038	0.418
Right side condyle superior space	0.355 ± 0.71	-0.215 ± 0.925	0.199
Left side condyle superior space	0.772 ± 0.91	0.143 ± 1.11	0.31
Right side condyle medial space	0.932 ± 1.47	1.158 ± 0.689	0.69
Left side condyle medial space	1.456 ± 1.32	1.116 ± 1.05	0.841
Right side condyle lateral space	0.122 ± 0.12	0.06 ± 0.306	1
Left side condyle lateral space	0.073 ± 0.19	0.197 ± 0.227	0.47
Right side condyle angulation *(degree)*	-1.83 ± 7.39	1.67 ± 3.08	0.574
Left side condyle angulation	7.67 ± 3.39	0 ± 3.1	0.013

## Discussion

The BSSO surgical technique involves splitting and repositioning the mandibular ramus bilaterally for optimal alignment. The predominant methods for reuniting the osteotomy fragments are fixation with bicortical screws or with monocortical screws and plates [8]. Despite these approaches, skeletal relapse remains a common post-BSSO complication [14]. Various screw and plate configurations have emerged to reduce post-surgery relapses and complications [15]. Recent focus has shifted to determining the fixation method that provides greater stability, while minimizing morbidity or complications [3].

In the present study, we evaluated the preoperative condylar position with immediate post-operative position. The similar short postoperative time period has been used by Kang et al. which evaluated the pre- and postoperative displacement and rotation of the condyle in the axial and sagittal plane to measure condylar position by sagittal split ramus osteotomy with and without bone graft. While short term postoperative evaluation of condylar changes could be one of our study limitations, cortical outlines may be more accurate to determine changes in short postoperative time but may not be valid to assess long-term changes because the cortical outlines can remodel with time and would no longer be reliable landmarks.

The current results indicated no statistically significant difference in condylar position parameters compared to preoperative measures within the lag screw fixation group. However, a marginally significant increase was observed in the left condyle’s angulation. It was in line with Harris et al.‘s findings [16], where BSSO advancements fixed with bicortical screws resulted in a medial angulation of the condyle. Angle et al. [17] reported statistically significant changes in transverse width and angulation of proximal segments in patients undergoing BSSO advancement with Le Fort I osteotomy. Besides, Alder et al. [18] assessed intercondylar angulations, examining condyles individually for angular changes using a midline reference’s perpendicular line. The study identified changes in all individuals within eight weeks.

The present study found no significant alterations in condylar position parameters compared to preoperative measures within the miniplate fixation group. Han and Hwang [19] compared fixation with a miniplate only, miniplate associated with single and multiple bicortical screws. The results showed that semi-rigid fixation using a miniplate yielded better recovery of condylar displacement compared to the hybrid technique.

The similarity in results between the two fixation methods in this study concurs with Kahnberg et al. [20], who found that both miniplates and lag screws resulted in negligible skeletal relapse, with no significant difference between the groups over up to 18 months of control. Similarly, Al-Moraissi et al.‘s [21] systematic review and meta-analysis revealed no statistically significant difference in skeletal stability between bicortical screw fixation and plate fixation of BSSO for mandibular setback. Sarkarat et al. [7] reported that the finite-element method indicated satisfactory primary stability using polymer-based resorbable screws and plates (polyglycolic acid and D, L-polylactide acid).

Sato et al. [22] detected that the skeletal stability was not significantly different among the patients undergoing mandibular advancement through any of the three rigid fixation methods of miniplates and monocortical screws, bicortical screws, and hybrid technique. Nor did they found any significant differences in the post-BSSO skeletal stability and condylar position between the sliding plate, miniplate, and bicortical screw fixation groups [23]. According to Yaripoor and Janbaz’s review article [24], using rigid fixation techniques subsequent to BSSO enhanced stability, yet the method of rigid fixation did not impact the stability. They declared that despite the widespread use of bicortical screws in the inverted-L position, this method would not enhance stability beyond other techniques.

Bohluli et al. evaluated biomechanical stress tolerance of screws used in 9 fixation methods after BSSO to identify a configuration imposing lower force on cortical bone at fixation points. They assembled a separated model utilizing 9 fixation methods: single screw, 2 screws arranged consecutively, 2 screws positioned vertically, 3 screws forming an L shape, 3 screws in an inverted backward L arrangement, miniplate with 2 screws, miniplate with 4 screws, 2 parallel plates (upper and lower border), and square miniplate with 4 screws. The study reported that although the most stable pattern was the inverted backward L configuration with three bicortical screws, all tested patterns provided adequate stability for clinical use [15].

In a computer modelling, Cox et al. [25] analyzed resorbable fixation and concluded that resorbable polymer-based plates and screws offer adequate strength and stiffness for mandibular angle fracture fixation. Furthermore, Tharanon [26] compared the biomechanical stability of three bicortical screws and a single four-hole miniplate following a 5-mm mandibular setback post BSSO in cadaver mandibles. They asserted that the two techniques were not significantly different in stability. However, a systematic review reported minimal discrepancies in skeletal stability between bicortical screws (titanium, stainless steel, or bioresorbable) and miniplates in the short term. The review also noted a higher number of studies depicting greater long-term skeletal relapse rates in patients treated with bicortical screws compared to miniplates [13].

Berköz et al. [27] suggested that despite the wide post-BSSO use of miniplate, single bicortical screw, and three bicortical screws, opting for a single screw in osteosynthesis might be advantageous when prioritizing the prevention of sagging. Conversely, Shetty et al. [28] contended that exclusively relying on miniplate fixation during sagittal split ramus osteotomies might not yield the consistent stability required for timely functional recovery. However, adding a retromolar positional screw significantly improves fixation stability in miniplate systems, providing technical and stability benefits over conventional miniplate or internal screw fixation.

A noteworthy finding from our study was the statistically significant increase in the angulation of the left condyle observed in the lag screw fixation group. While the absolute change in angulation (from 24.83° to 32.5°) may seem modest, this subtle shift in condylar positioning could have important clinical implications.

Alterations in condylar angulation, even within a relatively small range, have been associated with changes in mandibular kinematics and joint loading [29, 30]. Such changes in condylar orientation can potentially lead to asymmetries in masticatory function, joint stress distribution, and long-term remodeling of the temporomandibular joint [31].

In the context of BSSO procedures, the observed increase in left condyle angulation with lag screw fixation may predispose patients to a higher risk of postoperative temporomandibular joint issues, such as joint pain, clicking, or limited range of motion. These subtle changes in condylar positioning, if left unaddressed, could negatively impact the functional and aesthetic outcomes for patients.

The clinical significance of these findings is that surgeons should carefully consider the potential impact of the fixation method on condylar positioning when planning BSSO procedures. While both miniplate and lag screw techniques demonstrated overall stability in condylar position, the increased angulation observed with lag screws warrants further investigation to determine the long-term implications and whether it should influence the surgeon’s choice of fixation method for individual patients.

One limitation of the current study was the relatively small sample size. We recommend conducting further investigations with larger patient cohorts to corroborate the findings and enhance the statistical power and generalizability of the results. Besides, the retrospective design of the current study inherently limits the ability to control for potential confounding factors, such as variations in surgical techniques and radiographic imaging parameters across the patient cohort.

## Conclusions

The results of this study indicate that in BSSO procedures, employing either lag screw or miniplate fixation methods has no significant effect on condylar position parameters. The changes in condylar height, length, and width were not statistically different before and after BSSO in either the lag screw or miniplate groups.

While a marginally significant increase in left condyle angulation was observed in the lag screw group, the clinical relevance of this subtle change requires further investigation. Alterations in condylar angulation, even within a relatively small range, have been associated with potential changes in mandibular kinematics and joint loading, which could impact long-term functional outcomes. Overall, the findings suggest that both lag screw and miniplate fixation techniques can be effectively utilized in BSSO procedures without appreciable differences in the stability of condylar position. Consequently, the choice between these two rigid fixation methods can be made based on the surgeon’s preference, experience, and their assessment of the potential impacts on individual patient outcomes. Future prospective studies with larger sample sizes and longer follow-up periods would be valuable to confirm the comparative clinical implications of these two fixation techniques and their impact on temporomandibular joint health and function.

## Data Availability

The datasets used and/or analysed during the current study are available from the corresponding author on reasonable request.

## Abbreviations

BSSO: Bilateral sagittal split osteotomy
CT: Computed tomography
SD: Standard deviation
